# Habitat Availability Is a More Plausible Explanation than Insecticide Acute Toxicity for U.S. Grassland Bird Species Declines

**DOI:** 10.1371/journal.pone.0098064

**Published:** 2014-05-20

**Authors:** Jason M. Hill, J. Franklin Egan, Glenn E. Stauffer, Duane R. Diefenbach

**Affiliations:** 1 Pennsylvania Cooperative Fish and Wildlife Research Unit, Pennsylvania State University, University Park, Pennsylvania, United States of America; 2 USDA-ARS Pasture Systems and Watershed Management Research Unit, University Park, Pennsylvania, United States of America; 3 U.S. Geological Survey, Pennsylvania Cooperative Fish and Wildlife Research Unit, Pennsylvania State University, University Park, Pennsylvania, United States of America; University of Regina, Canada

## Abstract

Grassland bird species have experienced substantial declines in North America. These declines have been largely attributed to habitat loss and degradation, especially from agricultural practices and intensification (the habitat-availability hypothesis). A recent analysis of North American Breeding Bird Survey (BBS) “grassland breeding” bird trends reported the surprising conclusion that insecticide acute toxicity was a better correlate of grassland bird declines in North America from 1980–2003 (the insecticide-acute-toxicity hypothesis) than was habitat loss through agricultural intensification. In this paper we reached the opposite conclusion. We used an alternative statistical approach with additional habitat covariates to analyze the same grassland bird trends over the same time frame. Grassland bird trends were positively associated with increases in area of Conservation Reserve Program (CRP) lands and cropland used as pasture, whereas the effect of insecticide acute toxicity on bird trends was uncertain. Our models suggested that acute insecticide risk potentially has a detrimental effect on grassland bird trends, but models representing the habitat-availability hypothesis were 1.3–21.0 times better supported than models representing the insecticide-acute-toxicity hypothesis. Based on point estimates of effect sizes, CRP area and agricultural intensification had approximately 3.6 and 1.6 times more effect on grassland bird trends than lethal insecticide risk, respectively. Our findings suggest that preserving remaining grasslands is crucial to conserving grassland bird populations. The amount of grassland that has been lost in North America since 1980 is well documented, continuing, and staggering whereas insecticide use greatly declined prior to the 1990s. Grassland birds will likely benefit from the de-intensification of agricultural practices and the interspersion of pastures, Conservation Reserve Program lands, rangelands and other grassland habitats into existing agricultural landscapes.

## Introduction

The U. S. Geological Survey (USGS) North American Breeding Bird Survey (BBS) has documented that grassland birds declined faster than any other habitat guild of birds in North America over recent decades, including our study period of 1980–2003 [1], [2]. These declines have been largely attributed to habitat loss and degradation, particularly from an increasing scope and intensity of agricultural practices (the habitat-availability hypothesis) [3]–[5]. Total grassland losses from agriculture and other land uses have been dramatic – e.g., of >60 million ha of tallgrass prairie historically present in North America less than 14% remains in many areas [6]. While substantial acreage of grasslands were lost prior to the 1980s, native grassland in the tallgrass prairie region and elsewhere is still being lost to agriculture [6], [7], succession [8], and other land use changes [9]. Indeed, between 1982 and 1997 (the approximate timeframe of our study) the United States lost approximately 97,000 km^2^ of grasslands–primarily due to agricultural practices [6]. Intensive agricultural practices can also degrade habitat and adversely affect grassland bird populations via the overgrazing of grasslands, harvesting of forage crops earlier and more frequently, and through the conversion of heterogeneous rural landscapes into intensively-managed crop monocultures [10].

The influence of insecticides on grassland bird populations has received less attention than habitat loss, but researchers have long expressed concerns about excessive and widespread insecticide use [11], [12]. Pesticide use, especially insecticides, can poison and kill birds and reduce reproductive success (e.g., through eggshell thinning) and food supplies [10], [13], [14]. Recently, Mineau and Whiteside [15] evaluated possible correlates, including several measures of insecticide exposure, of declines in BBS trends of North American grassland birds. They reported that exposure to insecticides was more strongly correlated with grassland bird declines (the insecticide-acute-toxicity hypothesis) in the conterminous U.S. from 1980–2003 than several other indices of agricultural practices and habitat availability, such as herbicide use and changes in permanent pasture area. The study was highlighted in the *New York Times* [16] and other popular media, which demonstrates public concern for the declines of grassland birds and the impacts of insecticides on the environment. Mineau and Whiteside [15] concluded that toxic insecticides “offer a more plausible explanation for overall declines than does the oft-cited ‘habitat loss through agricultural intensification.’”

The conclusions of Mineau and Whiteside [15] have important implications for understanding grassland bird population dynamics, but these results are surprising for several reasons. While grassland birds have experienced substantial declines [1], [2] insecticide use in the U.S. has been substantially reduced over the past thirty years [17]. Alternative chemistries that replaced the most toxic organophosphates were available starting in the mid-1970s [18], and most of the reduction in insecticide use occurred prior to the 1990s [17]. Acute poisoning events have also declined over the last 20 years [19]. Meanwhile, substantial grassland loss to agricultural use has continued to occur over that same timeframe [7]. The resulting smaller and fragmented grasslands are more likely to harbor smaller and less diverse grassland bird communities because many grassland birds are area- and edge-sensitive species [4], [5], [20] that respond strongly to the configuration and amount of grasslands in agricultural landscapes [21], [22].

By suggesting that insecticide exposure is a more important correlate of grassland bird declines than changes in landscape structure and habitat availability, Mineau and Whiteside’s [15] analysis could have substantial implications for agricultural policy and practice. Therefore, we reassessed whether habitat availability or insecticide acute toxicity offers a more plausible explanation of grassland bird declines in North America. Our analysis differed in two important ways from that of Mineau and Whiteside [15]. Firstly, Mineau and Whiteside [15] reduced individual grassland species’ trends in each state to a dichotomous variable (1 = declining; 0 = increasing) and examined only two measures of habitat availability for grassland birds in agricultural landscapes. In contrast, we used the overall trend reported for the “grassland breeding” bird guild for each state [2], which accounts for the uncertainty among individual species’ trends within a state [23]. Secondly, we identified and evaluated several additional covariates representing habitat availability and reexamined the correlation between U.S. grassland bird declines (1980–2003), habitat availability, and insecticide use and acute toxicity.

## Methods

### North American Grassland Bird Data

The USGS North American Breeding Bird Survey is the only long-term continental monitoring program of North American breeding bird species. Each year volunteers across North America count breeding birds at 50 locations along 34.9-km roadside routes. We used the BBS “grassland breeding” species trends produced for each of 45 states between 1980 and 2003 (http://www.mbr-pwrc.usgs.gov/bbs/trend/guild03.html) - the same time period examined by Mineau and Whiteside [15]. These composite statewide trends incorporate any grassland bird species detected on ≥14 survey routes within a state, and account for uncertainty among individual species’ trends by adjusting them toward the mean trend of that state [23]. Additional details of BBS statistical procedure can be found in Link and Sauer [24], [25]. Some states with few grassland birds detected during BBS surveys (Alaska, Delaware, Hawai’i, Iowa, and Maryland) did not have state-wide trend estimates and hence were not included in the analysis.

### Agricultural Data and Covariates

We used publically available data primarily from the United States Department of Agriculture [USDA] Economic Research Service [26]–[28], the 1992 National Land Cover Data (NLCD [29]), the Natural Resources Conservation Service (NRCS [30]), and Mineau and Whiteside [15] to construct nine covariates describing agricultural intensity and landscape composition that we hypothesized would be relevant to grassland bird trends in the United States. Six of these covariates were used by Mineau and Whiteside [15], including the change in permanent pasture from 1978–2002, change in cropland for pasture from 1978–2002, agricultural intensity in 1992, agricultural herbicide use in 1992, agricultural insecticide use in 1992, and lethal insecticide risk to grassland birds in 1992. Change in permanent pasture or cropland for pasture, for each state, was calculated as the number of hectares in 2002 minus the number of hectares in 1978 divided by the number of hectares in 1978 [26], [28]. Permanent pasture was defined as land not used for arable crops and composed of primarily introduced forage plants managed for intensive grazing, as defined by the U.S. Department of Agriculture [USDA] Economic Research Service [26], [28]. In contrast, cropland for pasture (referred to as “cropped pasture” by Mineau and Whiteside [15]) is defined as land that was potentially suitable for arable crop production without improvements (e.g., adding tile drains or grading) but was used for short- or long-term livestock grazing rather than crop production in 1978 or 2002, respectively.

We indexed agricultural intensity as the percentage of all agricultural lands within a state allocated to active cropping (i.e., “cropland used for crops”) in 1992; data were obtained from the USDA Economic Research Service [27]. For pesticide use, we used the herbicide and insecticide indices presented in Mineau and Whiteside [15], which were calculated with data obtained from the National Pesticide Use Database maintained by the National Center for Food and Agricultural Policy (http://www.ncfap.org/database/state.php). This database provides estimates of areas of crops treated with individual pesticides. The insecticide and herbicide use indices were created with summed areas of crops within a state treated with each insecticide or herbicide, respectively, divided by the total areas of farmland within that state in 1992 [15]. In some states the herbicide index greatly exceeded 1.0, because many crops were treated with multiple herbicides. Unlike Mineau and Whiteside [15], we did not restrain herbicide use values at 1.0. Such an approach would have both underestimated herbicide use estimates and assumed equal herbicide exposure across 14 states. We used the lethal insecticide risk covariate presented in Mineau and Whiteside [15]. Briefly, lethal insecticide risk was taken to be the percentage of insecticide-treated farmland within a state over which logistic exposure models predicted some avian mortality for birds [18], [31]. State-specific estimates of pesticide use were only available in 1992 and 1997. Therefore, following Mineau and Whiteside [15], we used the lethal insecticide risk, agricultural intensity, herbicide use, and insecticide use data from only 1992 (the mid-point of the avian trend estimates). Thus our models, and those of Mineau and Whiteside [15], made the implicit assumption that these covariate values in 1992 were predictably related to other covariate values, such that states with high lethal insecticide risk in 1992 would have high insecticide risk in other years from 1980–2003.

Other herbaceous land cover types besides the two habitat types (permanent pasture and cropland for pasture) considered by Mineau and Whiteside [15] can benefit grassland birds [32], [33]. We defined three such covariates: statewide grassland coverage in 1992, Conservation Reserve Program (CRP) area in 1992, and change in rangeland from 1982–2002. We calculated the percentage of grassland coverage within a state from the “grassland” land cover classification from the 1992 NLCD [29], which excluded all grass or herbaceous cover within a state subjected to intensive management (e.g., residential lawns, golf courses, and crop fields). The NLCD categorization of grasslands includes grasslands (e.g., reclaimed surface mine grasslands and other non-grazed grasslands) not included in our other covariate categories. While percent change values for state-wide grassland variables could be more informative than a static value for 1992 grassland coverage, there are unfortunately no other national land cover estimates that cover the timeframe of our study.

The CRP covariate was expressed as the area (km^2^) of CRP lands within a state obtained from the NASS [34]. Change in rangeland was calculated as the rangeland area in 2002 minus the rangeland area in 1982 and expressed as a proportion of the 1982 area for each state using data from the NRCS [30]. Rangelands are extensively managed lands where the natural vegetation are mixtures of native grasses, forbs, and shrubs suitable for grazing as well as introduced forage species that have become naturalized [35].

### Statistical Analysis

We used an information theoretic approach to compare linear regression models with Akaike’s Information Criterion adjusted for sample size (AIC*_c_*). We separated our analyses into two suites of regression models to reduce model complexity and to enable a more direct comparison of our results with Mineau and Whiteside [15]. In the first suite of models we compared models created with the six covariates used by Mineau and Whiteside [15]. In the second suite of models we compared models created with lethal insecticide risk and the three additional covariates not considered by Mineau and Whiteside [15]. Our linear regression models took the general form of
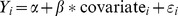
where 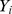 is the overall grassland bird trend (1980–2003) for state *i*, *α* is the global intercept for all states, *β* is the slope of the relationship for each covariate, and 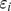 is the residual where 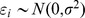.

Plots of the grassland bird trends and each covariate did not suggest any non-linear relationships, but at the suggestion of a reviewer we evaluated models with quadratic covariate terms. These models with quadratic terms yielded extraordinary large confidence intervals for coefficients (e.g., lethal insecticide risk) and hence we did not further consider such models. We examined residual plots from univariate linear regression models to determine if transformations of covariates were necessary. Based on these plots and the subsequent elimination of residual patterns, we square-root transformed the covariates representing agricultural intensity, lethal insecticide risk, insecticide use, herbicide use, and natural log (x+1) transformed the CRP area. We scaled and centered (i.e., standardized) all covariates to allow direct comparison of effect sizes among coefficients. Therefore, positive coefficients indicated a positive association with grassland bird trends and larger (absolute value) regression coefficients indicate a larger effect on grassland bird trends than coefficients closer to zero.

To reduce multicollinearity impacts [36], we excluded models from consideration (from all subsets of models) where combinations of predictors produced variance inflation factor (VIF) scores >5.0. In the first suite of models this criterion resulted in only models with ≤4 covariates and excluded models that contained both lethal insecticide risk and insecticide use. Lethal insecticide risk and insecticide use were correlated (*r* = 0.95, *P* = <0.001). At the suggestion of a reviewer we did evaluate models that contained both insecticide risk and insecticide use as predictors. Resulting coefficient estimates for these two covariates were highly uncertain with model-averaged 95% confidence intervals that were >4 times the width of the confidence intervals of any other covariate. Thus, correcting for multicollinearity allowed us to much more precisely estimate the effects of lethal insecticide risk. Consequently, we did not further consider models that included both insecticide use and insecticide risk. VIF scores did not exceed 5.0 for any of the models in the second model suite.

In the first model suite we considered 45 models (of all 56 possible models) that did not contain combinations of lethal insecticide risk and insecticide use; we considered all 16 possible models in the second set. When comparing models with AIC is it not uncommon for researchers to mistakenly assign importance to non-competitive models [37]. This often occurs when comparing a model with *K*+1 parameters to a better (i.e., lower AIC) nested model with only *K* parameters. The *K*+1 model may fall within 2 AIC units (ΔAIC≤2) of a simpler model, but such models “should not be interpreted as having any ecological effect” [37] unless they also substantially lower the model deviance [37], [38]. Therefore, after running both suites of models we then removed any nested model with one additional parameter (*K*+1) with an AIC*_c_* score greater than the simpler model with *K* parameters and a log-likelihood value <0.5 units smaller than the simpler model. We model-averaged coefficient estimates from the confidence model set (<4.0 ΔAIC*_c_* units from the top model [38]) and produced unconditional standard errors and 95% confidence intervals with the MuMIn package [39] in program R [40]. We model-averaged coefficient estimates using only the coefficient estimates from models that included that parameter of interest in the confidence sets.

## Results

### First Model Suite: Reevaluation of Mineau and Whiteside

**Figure 1:**
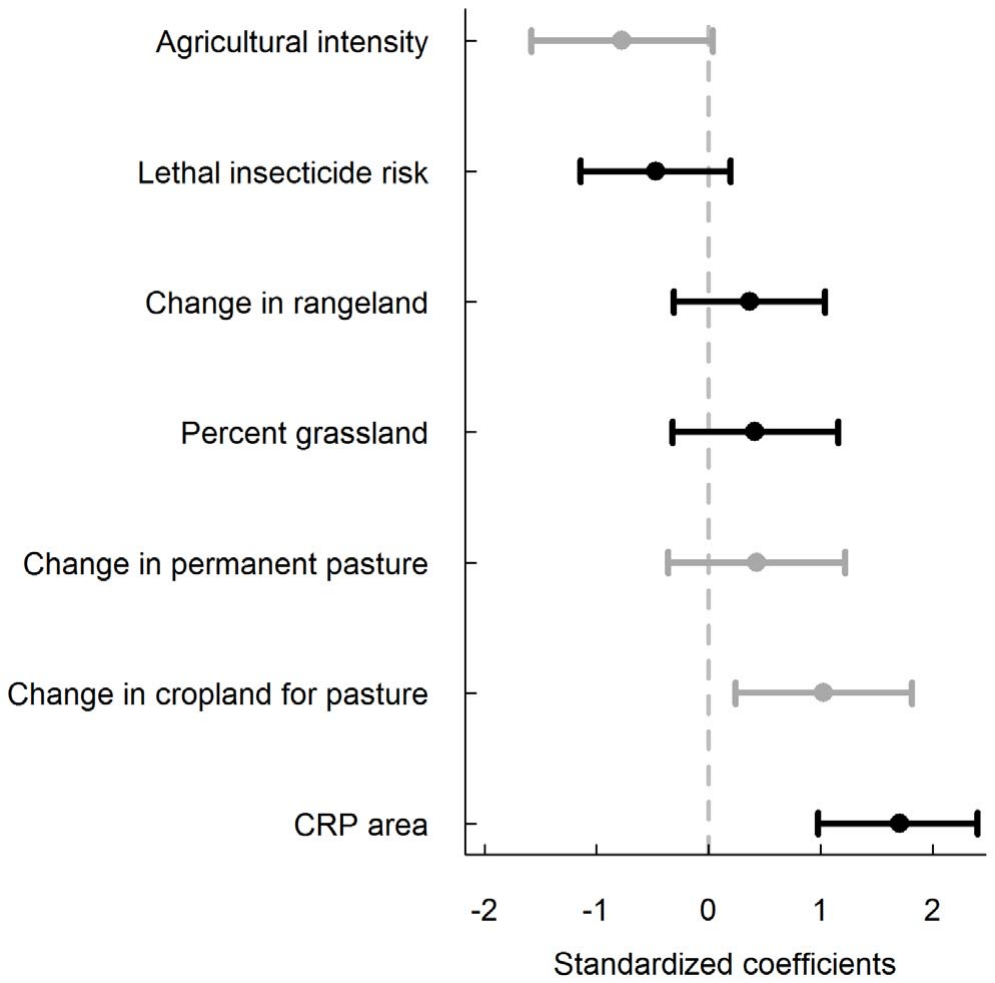
Model-averaged coefficients of covariates used to predict grassland breeding bird trends (1980–2003). Model-averaged coefficients (95% confidence intervals) occurring in the confidence sets of model suites one (gray bars) and two (black bars) that describe agricultural practices and habitat availability used to predict grassland breeding bird trends from 1980 to 2003 in the U.S.

Our model selection procedure from the first model suite resulted in 10 competitive models, three of which were included in the final confidence set (Table 1). However, only change in cropland for pasture had an estimated effect for which the confidence interval did not include zero (Figure 1). Change in cropland for pasture occurred in two of the three models in the confidence set (Table 1) and was positively associated with increasing bird trends (Figure 1). Only considering the best-performing models representing both hypotheses, habitat availability had 21.0 times more support (ratio of AIC***_c_*** weights = 0.42/0.02) than lethal insecticide risk as the most parsimonious explanation of the data (Table 1).

**Table 1:** Linear models from the first model suite with standardized coefficients^a^ that described the relationship between U.S. state grassland bird trends (1980–2003) and agriculture-related covariates (see Methods).

Change in cropland for pasture	Change in permanent pasture	Agricultural intensity	Herbicide use	Insecticide use	Lethal insecticide risk	*K* b	log(*L*)	AIC*_c_*	ΔAIC*_c_*	*w_i_* c
1.02						2	−106.87	220.32	0.00^d^	0.42
1.06	0.43					3	−106.27	221.54	1.21^d^	0.23
		−0.77				2	−108.25	223.08	2.76^d^	0.11
	0.43	−0.82				3	−107.68	224.37	4.05	0.06
		−1.36	0.71			3	−107.74	224.49	4.16	0.05
				−0.49		2	−109.32	225.22	4.90	0.04
			0.41			2	−109.53	225.64	5.32	0.03
		−1.53	1.27	−0.63		4	−107.06	225.66	5.34	0.03
	0.33					2	−109.69	225.97	5.64	0.03
					−0.27	2	−109.79	226.16	5.83	0.02

a Model-averaged coefficients with unconditional standard errors: change in cropland for pasture (*β* = 1.03, SE = 0.40), change in permanent pasture (*β* = 0.43, SE = 0.40), and agricultural intensity (*β* = −0.77, SE = 0.41).

b No. parameters.

c Akaike model weights.

d Models appearing in the confidence set.

### Second Model Suite: Lethal Insecticide Risk & Additional Habitat Covariates

For the second model suite, our model selection procedure resulted in nine competitive models, six of which were included in the confidence set. All four possible covariates were included in the confidence set (Table 2), but again, confidence intervals for the estimated effect of most covariates included zero. CRP area had the largest effect size, was positively associated with grassland bird trends, and was the only covariate that had an estimated effect for which the confidence interval did not include zero (Figure 1). We had originally expressed the CRP covariate as the change in CRP area over time within a state, but we changed it to its current form at the request of a reviewer–our results and conclusions did not change. Although included in the confidence set, change in rangeland, percent grass, and lethal insecticide risk all had uncertain effects on grassland bird trends (Table 2, Figure 1). The most parsimonious model included only CRP area (Table 2), and this model had >1.3 times more support (ratio of AIC***_c_*** weights = 0.28/0.22) than the highest ranking model containing lethal insecticide risk (Table 2). Comparing only univariate models, the CRP model had >2300 times more support than the lethal insecticide risk model. CRP had approximately 3.06 times the effect (ratio of standardized regression coefficients = 1.71/0.47) on grassland bird populations than did lethal insecticide risk.

**Table 2:** Linear models with standardized coefficients^a^ that appeared in the second model suite that described the relationship between U.S. state grassland bird trends (1980–2003), lethal insecticide risk, and three additional habitat covariates not considered by Mineau and Whiteside (2013).

CRP area	Percent grassland	Change in rangeland	Lethal insecticide risk	*K* b	log(*L*)	AIC*_c_*	AIC*_c_*	*w_i_* c
1.71				2	−99.71	206.01	0.00^d^	0.28
1.76			−0.47	3	−98.73	206.46	0.45^d^	0.22
1.52	0.42			4	−99.08	207.15	1.14^d^	0.16
1.78		0.36		3	−99.15	207.30	1.28^d^	0.15
1.84		0.39	−0.49	4	−98.05	207.64	1.63^d^	0.12
1.59	0.42	0.36		4	−98.49	208.53	2.51^d^	0.08
	1.09			2	−106.34	219.28	13.26	<0.01
			−0.27	2	−109.79	226.16	20.15	<0.01
		0.02		2	−110.00	226.58	20.57	<0.01

a Model-averaged coefficients with unconditional standard errors: CRP area (*β* = 1.71, SE = 0.37), percent grassland (*β* = 0.42, SE = 0.38), change in rangeland (*β* = 0.37, SE = 0.35), and lethal insecticide risk (*β* = −0.47, SE = 0.34).

b No. parameters.

c Akaike model weights.

d Models appearing in the confidence set.

## Discussion

Our results indicate that population trends of grassland birds in the U.S. are primarily associated with habitat availability, rather than insecticide use or insecticide acute toxicity. Thus, in direct contrast to Mineau and Whiteside [15], our results do not support the insecticide-acute-toxicity hypothesis because we found stronger connections with declines in grassland bird abundance using habitat-based covariates. Pesticides may negatively influence grassland birds, but our results strongly support habitat associations over insecticide-acute-toxicity as the more plausible explanation for observed BBS trends.

We suggest three reasons why we arrived at different conclusions than Mineau and Whiteside [15]. First, Mineau and Whiteside’s [15] use of a dichotomous response variable based on the point estimate of a species’ trend means that species with steeply negative or precisely estimated trends are treated identically to species with near-zero or poorly estimated trends. In contrast, we used a single state trend for grassland bird species which explicitly accounted for the similarity and uncertainty among all grassland bird trends within that state [23]. Second, Mineau and Whiteside [15] analyzed individual grassland species’ trends (1 = decreasing, 0 = increasing) without accounting for the lack of independence among trends from within a state (e.g., by treating state identity as a random effect). Trends of grassland birds from within a state are unlikely to be independent of one another due to similar land management, agricultural policies, pesticide exposure, weather conditions, and habitat availability that affect all species within that state. Failure to account for this lack of independence would not necessarily favor insecticide acute toxicity in the modeling process, but the violation of basic linear model assumptions can result in spurious results [41]. Third, we observed evidence of the effects of multicollinearity in Mineau and Whiteside’s [15] analysis. For example, when insecticide use and lethal insecticide risk occurred within the same model (16 such occurrences in Mineau and Whiteside [15]) the coefficient for insecticide use was always negative, whereas it was always positive when it occurred in models without lethal insecticide risk (16 occurrences) (JMH unpublished data). Multicollinearity can complicate model selection, result in large variances, and cause coefficient estimates to reverse sign when another co-linear variable is included in the model [36], [42]–[44]. We reduced multicollinearity effects by limiting model selection to combinations of linear predictors where VIF scores were <5.0.

Consistent with the prediction of the habitat-availability hypothesis we found that grassland bird trends were more strongly associated with (i.e., larger effect sizes of standardized coefficients) CRP area, agricultural intensity, and trends in cropland for pasture than they were for lethal insecticide risk (Tables 1 and 2). These results are consistent with the continuing decline of grassland birds over the study period [2] and the dramatic conversion of rangelands, pasturelands, and native grasslands to intensive croplands and developed land over the last 35 years [7], [30]. For example, cropland for pasture area in the U.S. declined by 20% (6.3 million ha) from 1978 to 2002 (approximately the time frame of this study) and declined by a further 41% (10 million ha) from 2002 to 2007 alone [30], [45]. Meanwhile overall pesticide use in the U.S. has likely remained relatively stable and insecticide use has likely declined since the 1980s [17]. Additionally, several researchers have compared the influence of habitat availability relative to the prevalence of organic (i.e. pesticide free) cropland. These studies have consistently found that the extent and configuration of grassland habitats has stronger effects on the richness and abundance of most grassland bird species than the amount of organic acreage [46]–[48]. As grassland area declines in agricultural landscapes the potential for negative impacts to grassland bird populations increases because many grassland bird species are sensitive to fragmentation and area and edge effects [4], [5], [20], [49], [50].

Our models suggest that grassland birds respond negatively to agricultural intensification [32], [51] (i.e., increases in row-crop agriculture), but less-intensive agricultural practices may benefit some grassland bird species [52]. For example, light grazing pressure, and rangelands in general, have been positively linked to abundances of many species of grassland birds such as grasshopper sparrows (*Ammodramus savannarum*), Henslow’s sparrows (*A. henslowii*), and upland sandpipers (*Bartramia longicauda*) [9], [53]–[55]. Some grassland birds breed in hayfields and pasturelands [32], [56], and adult bobolink (*Dolichonyx oryzivorus*) and savannah sparrow (*Passerculus sandwichensis*) apparent survival is negatively related to the intensity of agricultural practices in these hayfields [57]. The value of hayfields to breeding grassland birds, however, may be dependent upon the timing of mowing activities [58], [59]. The point estimate for the effect of permanent pasture on grassland bird trends was positive, but the effect was uncertain and smaller than the effect of change in cropland for pasture area (Figure 1). This result may be related to the relatively small amount of permanent pasture changes observed across states from 1978 to 2002 (mean = −647 ha) compared to cropland for pasture changes (mean = −120,880 ha) over the same time period [26], [28].

We found that CRP area was the strongest predictor (i.e., largest standardized coefficient; Figure 1) of grassland bird trends in the U.S. Other researchers have also documented positive effects of CRP area on some species of grassland birds [33], [60], [61], but see Pabian et al. [62]. We found nearly identical results when we expressed the CRP variable as the change in percent of acreage over time within a state (JMH unpub.). The Conservation Reserve Program is a type of agricultural de-intensification where farmers are paid to remove environmentally sensitive lands from agricultural production. However, as farmland values have risen sharply over the last decade [63], CRP enrolled hectares have dropped by 29% from 2006 to 2013 [64], [65]. Our results suggest that CRP program enrollment may be an important component of grassland bird conservation strategy in the U.S. However, while CRP and other restored or non-native grasslands may benefit some species, these areas may not provide optimal habitat for some grassland bird species (e.g., Sprague’s pipt [*Anthus spragueii*], and short-eared owl [*Asio flammeus*]) that are relatively intolerant to anthropogenic disturbance ([66], [67]). The best conservation strategy for such species may be to protect remaining native grasslands from conversation to agricultural lands [5], [68], [69].

Our results are consistent with the scarcity of data linking pesticides to vertebrate population changes [19], but depend on several important assumptions. Following Mineau and Whiteside [15] we developed several covariates based on data from 1992 (the midpoint of our study period), because data for these covariates was not available circa 1980; thus our models assume that these 1992 covariate values were predictably related to other years in our analysis. Insecticides have direct and indirect negative effects on many groups of birds [12], [14], [70], [71], but the phase-out of especially toxic active pesticide ingredients (e.g., organophosphates and cholinesterase-inhibiting carbamates) combined with the increased use of Bt crops, and reduced application rates of insecticides [17], [18] may have lowered the risk of lethal avian exposures to North American grassland bird species over the past 20 years. If overall insecticide risk to grassland birds declined non-linearly between 1992 and 2003 then both we and Mineau and Whiteside [15] may have imprecisely estimated the risk of insecticides to grassland birds. Our models suggested that insecticide acute toxicity was negatively related to grassland bird trends, but insecticide acute toxicity was a relatively poor predictor of grassland bird trends on its own (Table 1). Our analyses could be improved if finer scale (temporal and spatial resolution) land use, pesticide application, and pesticide exposure data were available for the conterminous United States from 1980–2003.

Our results and those of others [15], [32], [33] that are based on broad-scale correlations of BBS and agricultural data clearly cannot prove or disprove the insecticide-acute-toxicity or habitat-availability hypotheses. However, the strong majority of the grassland bird research [5], [20], [61], [72]–[76] suggests that grassland bird populations in the U.S. are most strongly related to grassland configuration and amount (the habitat-availability-hypothesis). Further grassland bird population declines are foreseeable given the continued loss of U.S. grasslands [7], [30], [45]. Building multifunctional landscapes that provide an abundant food supply while also conserving biodiversity and supplying ecosystem services, has become a central challenge for agriculture in the 21st century [77], [78]. Farmers, conservation organizations, and governments have taken many approaches to build multifunctional working landscapes [79]–[82]. Our results, and those of others [5], [61], [72] suggest that an approach most effective for grassland bird conservation would include a focus on habitat creation and preservation, encouraging CRP acreages, pastures, and low-intensity grazing land within intensive agriculture landscapes, and strongly disincentivizing the conversion of remaining native grasslands to agricultural lands.
